# Oxidative Stress and Redox-Dependent Pathways in Cholangiocarcinoma

**DOI:** 10.3390/antiox13010028

**Published:** 2023-12-22

**Authors:** Alessandra Caligiuri, Matteo Becatti, Nunzia Porro, Serena Borghi, Fabio Marra, Mirella Pastore, Niccolò Taddei, Claudia Fiorillo, Alessandra Gentilini

**Affiliations:** 1 Department of Experimental and Clinical Medicine, University of Florence, 50139 Florence, Italy; alessandra.caligiuri@unifi.it (A.C.); fabio.marra@unifi.it (F.M.); mirella.pastore@unifi.it (M.P.); 2 Department of Experimental and Clinical Biomedical Sciences “Mario Serio”, University of Florence, 50139 Florence, Italy; matteo.becatti@unifi.it (M.B.); nunzia.porro98@outlook.it (N.P.); serena.borghi@unifi.it (S.B.); niccolo.taddei@unifi.it (N.T.)

**Keywords:** cholangiocarcinoma, redox, ROS, RNS, inflammation, oxidative damage, nitrosative damage

## Abstract

Cholangiocarcinoma (CCA) is a primary liver tumor that accounts for 2% of all cancer-related deaths worldwide yearly. It can arise from cholangiocytes of biliary tracts, peribiliary glands, and possibly from progenitor cells or even hepatocytes. CCA is characterized by high chemoresistance, aggressiveness, and poor prognosis. Potentially curative surgical therapy is restricted to a small number of patients with early-stage disease (up to 35%). Accumulating evidence indicates that CCA is an oxidative stress-driven carcinoma resulting from chronic inflammation. Oxidative stress, due to enhanced reactive oxygen species (ROS) production and/or decreased antioxidants, has been recently suggested as a key factor in cholangiocyte oncogenesis through gene expression alterations and molecular damage. However, due to different experimental models and conditions, contradictory results regarding oxidative stress in cholangiocarcinoma have been reported. The role of ROS and antioxidants in cancer is controversial due to their context-dependent ability to stimulate tumorigenesis and support cancer cell proliferation or promote cell death. On these bases, the present narrative review is focused on illustrating the role of oxidative stress in cholangiocarcinoma and the main ROS-driven intracellular pathways. Heterogeneous data about antioxidant effects on cancer development are also discussed.

## 1. Introduction

### 1.1. Cholangiocarcinoma

Cholangiocarcinoma (CCA) arises from malignant transformation of the biliary epithelial cells. It can be anatomically classified as intrahepatic (iCCA) or extrahepatic (eCCA), the latter being further subdivided into perihilar (pCCA) and distal CCA (dCCA), depending on the site of onset within the biliary system [1,2]. CCA is a fatal tumor, accounting for 2% of all cancer-related mortalities worldwide yearly due to its high aggressiveness and poor response to current therapies. Furthermore, over the past few decades, CCA mortality has increased globally [3,4].

Mostly asymptomatic in the early phases, it is diagnosed at an advanced state of disease when therapeutic treatments are inefficient, and only a few CCA patients qualify for potentially curative surgical procedures [3,4].

Although the pathogenesis of CCA is still largely unknown, this tumor arises in a background of chronic hepatobiliary inflammation. Numerous risk factors have been associated with its onset, especially for iCCA, including primary sclerosing cholangitis (PSC), alcohol abuse, chronic hepatitis B and C, cirrhosis, diabetes, and nonalcoholic fatty liver disease (NAFLD), even if most CCAs have no recognizable cause [4]. All these risk factors are characterized by a persistent inflammatory condition associated with an overproduction of pro-inflammatory cytokines and reactive oxygen and nitrogen species (RONS). These molecules are responsible for the creation of a pro-oxidant environment, resulting in chronic injury to the biliary tract [5].

### 1.2. ROS, RNS, and Antioxidants in Cancer

Reactive oxygen species (ROS) and reactive nitrogen species (RNS) are highly reactive molecules and ions containing oxygen or nitrogen, respectively. They are produced naturally in cells as byproducts of metabolism and are recognized for their role in cellular signaling, playing a dual role in cancer [6]. In the context of cancer, RNS, along with ROS, can participate in oxidative and nitrosative stress, leading to the damage of cellular components, such as DNA, proteins, and lipids, and contributing to the initiation and progression of cancer. Additionally, RNS may influence processes such as angiogenesis and immune responses, which are important for tumor growth and survival. The relationship between RNS and cancer is complex, and the context-specific effects of RNS can vary depending on the type of cancer, stage of cancer development, and other factors [7].

Oxidative stress displays a “double-edged sword” effect on the occurrence and development of tumors. On the one hand, ROS can activate growth-promoting signals, enhance the proliferation and invasion of tumor cells, and cause damage to biomolecules; on the other hand, ROS can enhance antitumor signaling pathways by initiating oxidative stress-induced apoptosis and autophagy in cancer cells [8].

In cancer, hydrogen peroxide (H_2_O_2_) is a well-studied signaling molecule [9] produced by the enzymatic action of superoxide dismutases 1, 2, and 3 (SOD1, 2, and 3) situated in the cytosol, mitochondrial matrix, and extracellular space, respectively. H_2_O_2_ facilitates signal transduction by oxidizing cysteine residues within proteins, thereby modifying their functions [10]. The superoxide ion (O_2_^−^) has also been identified as participating in redox signaling and the initiation of cell death [11]. Additionally, H_2_O_2_ in Fenton chemistry (with ferrous or cuprous ions) generates hydroxyl radicals (OH^•^) capable of molecular damage. Antioxidants such as peroxiredoxins (PRXs), glutathione peroxidases (GPXs), and catalase (CAT) convert intracellular H_2_O_2_ into water (H_2_O) and O_2_ [12].

Cancer cells adapt to high ROS levels by activating antioxidant pathways, increasing ROS clearance [13]. Elevated endogenous ROS levels in cancer cells heighten sensitivity to ROS-inducing therapy, as many chemotherapeutics boost ROS production [14]. Inducing ROS emerges as a viable strategy against cancer. To maintain redox balance, cells activate the Nrf2 transcription factor [14,15], which, under high ROS levels, induces the expression of antioxidant genes like GPXs, PRXs, and CAT involved in glutathione (GSH) synthesis/utilization and detoxification [16]. Nrf2 activation also stimulates NADPH production by regulating enzymes in the pentose phosphate pathway (PPP) and serine biosynthesis pathways [17]. Crucial for cancer cell proliferation and tumorigenesis, Nrf2 loss inhibits tumor burden by promoting oxidative stress-induced cancer cell death [18].

Also, tumor suppressors play important roles in terms of antioxidant defense in normal cells. BRCA1 stabilizes and activates its partner Nrf2, preventing ROS accumulation [17]. Mitochondrial SIRT3 deacetylates SOD2 to regulate its scavenging function. In the absence of SIRT3, reduced SOD2 activity leads to ROS accumulation, stabilization of the hypoxia-inducible factor-1α (HIF-1α) protein, and the onset of tumorigenesis [19].

The tumor suppressor p53 regulates the expression of several antioxidant genes, including SOD2, GPX1, and CAT. When p53 is lost or mutated (occurring in over 50% of human cancers), the ROS level increases, enabling cellular transformation and protumorigenic signaling [20]. The delicate balance between ROS production and removal is crucial to maintaining an optimal ROS level able to enhance protumorigenic signaling.

### 1.3. Ros Signaling and Tumorigenesis

ROS are able to promote tumorigenesis [21]. The mutated oncogene Ras increases NOX4-derived ROS production, thus enhancing proliferation [22]. ROS can hyperactivate the phosphoinositide-3-kinase-PI3K/Akt/mTOR survival pathway [23]. Furthermore, the oncogenic activation of Akt can increase ROS production to further promote cancer cell survival and proliferation [24]. ROS also oxidize and inactivate Mitogen-activated protein kinases (MAPK) phosphatases, inducing growth factor receptor activation and MAPK/ERK pro-proliferative signaling and can facilitate tumor cell survival by activating NF-κB and Nrf2, transcription factors that upregulate the expression of antioxidants [25].

ROS have been reported to be also implicated in promoting cancer metastasis and angiogenesis [26]. When highly proliferative tumors exceed their blood supply, regions within the solid tumor become hypoxic and glucose-deprived, enhancing ROS production. Then, cancer cells undergo metabolic changes to increase NADPH production and maintain redox homeostasis [27]. ROS contribute to metastasis through the stimulation of several mechanisms, such as the PI3K regulatory subunit/AkT serine/threonine kinases/mechanistic target of rapamycin kinase (PI3K/Akt/mTOR), MAPK signaling pathways, which activate downstream the transcription factor SNAIL, MMP2 (metalloproteinase 2), and MMP9 (metalloproteinase 9) enzymes initiating epithelial-mesenchymal transition (EMT) leading to metastasis [28]. Furthermore, several studies have indicated ROS to be a major cause of epithelial to mesenchymal transition (the major cause of tumor metastasis), a process where epithelial cells lose their polarity, cell–cell adhesion, and gain mobility.

It has also been shown that ROS are responsible for evoking antitumorigenic signaling [29]. ROS promote cell death through the activation of the ASK1/JNK and ASK1/p38 signaling pathways [30]. Inactivating mutations in the p38 and JNK signaling pathways have been reported in numerous human tumors, suggesting that these pathways promote cancer cell death [31,32].

## 2. Interaction between Oxidative Stress and Inflammation Focused in CCA

In recent years, it has emerged that the reciprocal action between persistent oxidative stress and prolonged inflammation plays an important role in tumorigenesis, especially regarding cancer initiation [33,34].

In inflamed liver, a wide variety of soluble mediators are released by injured hepatocytes or cholangiocytes and recruited/activated inflammatory cells, able to regulate cell behavior. By binding to the cognate receptors on cell surfaces, these factors elicit a cascade of intracellular events that control cell growth and survival, cell motility, metabolic reprogramming, and secretory properties. On the other hand, oxidative stress is also present in inflammatory sites. High amounts of RONS, released by both injured epithelial cells and inflammatory cells, bind and damage proteins, lipids, and nucleic acids, disrupt cellular membranes, and induce DNA lesions and mutations. This process further sustains inflammatory response, giving origin to a vicious circle in which persistent oxidative stress and long-lasting inflammation amplify each other and create an environment that favors cancer initiation and progression [35]. Along these lines, it is not surprising that DNA lesions induced by oxygen or nitrogen species have been specifically found in tumors developed on a background of chronic infection or inflammation [36].

In the presence of persistent inflammatory stimuli, infiltrating and resident (primarily Kupffer cells, KCs) inflammatory cells respond by secreting a variety of mediators that, in addition to recruiting other immune cells, activate diverse cell types, including hepatic stellate cells (HSCs), fibroblasts, and endothelial cells that go through a series of phenotypic changes. As a consequence, sustained inflammation can give origin to a rearranged microenvironment favoring cancer onset. In this setting, the cancerogenic process can also be facilitated by the inhibitory actions exerted by some soluble factors, such as TGFβ, VEGF, TNFα, IL1, and IL6, on immune response [37]. In CCA, as in other desmoplastic tumors characterized by a highly reactive stroma, activated stromal cells (mainly cancer-associated fibroblasts, CAFs, and tumor-associated macrophages, TAMs) act in concert with a wide variety of noncellular components (extracellular matrix molecules and soluble factors) to promote cancer progression [35,38]. In this context, a plethora of cytokines (TGFβ, TNFα, IL1, IL6, etc.) and chemokines (IL8, CCL2, CXCL12, etc.) mediate the crosstalk between injured epithelial cells and stromal components. Beyond their pro-inflammatory action, these agents are able to induce intracellular pathways and activate or repress a variety of transcription factors (such as NF-κB, HIF-1α, STAT3, AP1, Nrf2, and Wnt/β-catenin) that control main cellular processes and, if dysregulated, may lead to neoplastic transformation [33,34]. Among these, a crucial role is played by NF-κB, which upregulates several pro-inflammatory genes, including TNFα, IL1, and IL6, but has also been shown to act as a tumor promoter in inflammation-related cancers [39]. Moreover, under inflammatory stimuli (such as LPS, TNFα, IL1, and IFNγ), both inflammatory and epithelial cells express high levels of inducible nitric oxide synthase (iNOS) that synthesizes large amounts of NO, an unstable and highly reactive molecule triggering nitrative cell damage and tumorigenic potential [40,41]. Notably, iNOS expression can be induced upon activation of NF-κB. Cyclooxigenase-2 (COX-2) is also activated during sustained inflammation by TNFα and IL6, leading to ROS and RNS accumulation [41]. In this environment, free radicals and reactive metabolites are abundantly released and accumulate, causing further cell damage and potentiating inflammation. In addition to directly affecting cell components and inducing DNA damage and genomic instability, reactive intermediates are also able to act as second messengers, stimulating pro-inflammatory pathways and modulating transcription factors such as NF-κB, AP1, Wnt/β-catenin, and p53. As a consequence, in a chronic inflammatory environment, oxidative/nitrative stress and inflammatory mediators concur with cancer development by influencing major biological processes, such as apoptosis, proliferation, migration, angiogenesis, oxidative stress resistance, and metabolic changes.

The interplay between oxidative stress and inflammatory factors in promoting CCA has been investigated by Yuan et al., who showed that KC-derived inflammatory signals play a crucial role in CCA initiation under elevated oxidative stress and mitochondrial dysfunction. In their iCCA models, KCs released high amounts of tumor necrosis factor (TNF) that, through a paracrine action mediated by TNFR/JNK/c-jun, promoted cholangiocyte overgrowth and neoplastic transformation. These findings were in accord with data observed in human iCCA specimens in which high levels of oxidative stress-induced DNA lesions in the tumor and adjacent tissues correlated with increased expression of TNF around the tumor and of pJNK in CCA cells. Of note, the abundance of ROS-induced DNA alterations found in hepatocytes surrounding the tumor, other than in malignant cholangiocytes, indicated that oxidative stress in the tumor microenvironment highly contributes to CCA development. In agreement with these observations, the treatment of the animals with antioxidants, depletion of KCs, deletion of TNF receptor (TNFR), or inhibition of JNK resulted in reduced tumorigenesis. In addition, liver-specific knockout of JNK1/2 was associated with lower tumor progression and improved survival rate [42].

In the context of chronic inflammation, oxidative/nitrative stress can even act as an inducer of cancer stem cell niche formation. In inflamed livers of CCA patients infected by *Opistorchis viverrini*, Kawanishi et al. found several OV6+/CD133+ progenitor/stem cells harboring high levels of 8-oxodG (8-oxo-7,8-dihydro-2′-deoxyguanosine), a marker of free-radical-induced DNA damage. This evidence suggests that proliferating progenitor cells, feasibly activated to regenerate disrupted bile ducts, could be targeted by reactive species abundantly released in that area and go through DNA lesions/mutations, eventually leading to the onset of a cancer stem cell niche [43].

Moreover, the recent literature data reported the involvement of RONS in the modulation of specific long noncoding RNAs (lncRNAs) that regulate inflammatory processes. In particular, short-term and long-term oxidative stress has been shown to induce the up-regulation of two lncRNAs, HULC and H19, in cholangiocyte cell lines exposed to glucose oxidase. The stimulation of H19 and HULC correlated with the expression of pro-inflammatory genes, suggesting that lncRNAs might promote CCA tumorigenesis, mediating the positive feedback between inflammation and oxidative stress [44].

Finally, among the factors implicated in inflammation-related tumorigenesis is insulin receptor substrate 1 (IRS1), an adaptor protein involved in insulin signaling mediated by both insulin and insulin-like growth factor receptors. IRS1 has been shown to play a role in the link between inflammation and oxidative stress in several tumors (breast, prostate, pancreatic, etc.), including CCA. In recent work, Kaewlert and colleagues showed that IRS1 was overexpressed in malignant cells of CCA patients in association with the oxidative stress marker 8 oxodG, and this was predictive of poor prognosis. This observation was also confirmed in vitro by the same authors, who compared immortalized cholangiocytes (MMNK1), immortalized cholangiocytes subjected to long-term oxidative stress (ox MMNK1 L) to induce oxidative stress resistance, and different CCA cell lines, detecting an increased expression of IRS1 in oxidative stress resistant ox MMNK1 L and CCA cells respective to immortalized cholangiocytes. Notably, the downregulation of IRS1 in CCA cells resulted in a decrease in proliferation and block of cell cycle progression, together with a reduction of motility, invasiveness, oxidative stress resistance, and stemness features. Accordingly, several genes and intracellular pathways regulating these processes (MAP3K3, YAP1, vimentin, TGFβR, Nrf2, and others) were downregulated as well [45].

## 3. CCA and Oxidative Damage

Increased levels of intracellular ROS can directly oxidize and damage DNA, inducing genome instability and mutation. The most susceptible base subjected to oxidative damage is guanine, which can be converted to 8-hydroxy-2′-deoxyguanosine (8-OH-dG), resulting in point mutations if not properly repaired by base excision repair (BER) enzymes or nucleotide excision repair (NER) enzymes. The literature data reveal an increased expression of 8-OH-dG in CCA patients [46,47,48]. A study has particularly focused on the formation of oxidative DNA lesions in cholangiocarcinoma tissues in relation to stem/progenitor cell marker expression, showing a higher accumulation of 8-oxodG in CD133- and/or Oct3/4-positive tissues [49].

Another consequence of high ROS levels is lipid damage. ROS can trigger lipid peroxidation, an oxidative chain reaction in which oxidants attack lipids containing carbon-carbon double bonds, especially polyunsaturated fatty acids (PUFAs). Being highly reactive compounds, lipid peroxides can propagate further the generation of ROS and dissociate into compounds capable of forming crosslinks with DNA and proteins. Higher oxysterol levels have been found in human bile in relation to biliary tract infection and inflammation, highlighting their pathophysiologic role in the development and progression of CCA. In particular, the oxysterol 22-HC seems to stabilize COX-2 mRNA through a p38 MAPK-dependent mechanism, resulting in COX-2 accumulation in a CCA cell line [50]. COX-2 has been shown to be highly expressed in CCA, and its involvement in CCA progression may be related to prostaglandin E_2_ (PGE_2_) production, which stimulates cell proliferation and angiogenesis and inhibits apoptosis [51].

Impaired redox status in CCA patients was confirmed by the presence of oxidative stress-induced protein carbonylation in tumor tissues, which leads to loss of specific protein function. In particular, CCA tissues showed high carbonylation levels of serotransferrin and other iron-binding proteins, such as ferritin and albumin, leading to protein aggregation and iron accumulation. CCA tissues with carbonylated serotransferrin show significantly higher Fe^3+^ concentrations than those with noncarbonylated serotransferrin [52,53]. Iron can contribute to both tumor initiation and progression. Excess iron can lead to ROS formation through Fenton reaction and mutagenesis, as shown by an increased cancer risk in patients with iron overload. Different expressions of iron proteins, indicating increased iron content, have been detected in human intrahepatic CCA cell lines (CCA4, CCLP1, and HUCCT1). The differences in iron levels were accompanied by changes in oxidative stress: both the expression of heme oxygenase (HO-1), a well-known marker of oxidative stress, and ROS levels have been found to be remarkably higher in untreated CCA cell lines [54]. In addition, chronic iron overload may enhance iNOS activity since heme iron is required for dimerization and activation of this enzyme, which is responsible for elevated NO production [55].

Recent data show that cellular senescence may be involved in the pathophysiology of CCAs [56,57]. Cell senescence is characterized by proliferative arrest, which is mediated by complex mechanisms, such as epigenetic modifications, genomic instability, DNA damage, chronic inflammation, and chromatin remodeling [58]. In the development of cholangiocarcinogenesis, oncogene- or DNA damage-induced senescence may occur, acting as a potent antitumor mechanism, and escaping from it is essential for CCA onset [59,60]. Nevertheless, senescent cells in precursor lesions and cells around cholangiocarcinoma may play important roles in tumor development and progression via secretion of the senescence-associated secretory phenotypes (SASPs) consisting of biologically active factors, including chemokines, interleukins, and proteases [58].

The evidence shows that reactive oxygen molecules can contribute to the induction of cellular senescence [61,62]. Indeed, it has been observed that inflammatory cytokines generated ROS in cultured biliary epithelial cells, followed by activation of the ATM/p53/p21WAF1/Cip1 pathway leading to cellular senescence [63].

Furthermore, it has been shown that the polycomb group protein Bmi1, which causes a decrease in p16INK4a, may be involved in cellular senescence. In cultured biliary epithelial cells, oxidative stress significantly decreased Bmi1 expression, followed by increased p16INK4a levels with consequent augmentation of cellular senescence [61].

## 4. CCA and Nitrosative Damage

NO generation by iNOS is triggered during infection and inflammation. Overproduction of NO is linked to nitrative DNA and protein damage, resulting in the formation of 8-nitroguanine and carcinogenic N-nitrosamines. 8-nitroguanine formation occurs to a greater extent in CCA tissues than in noncancerous tissues, and its spontaneous depurination leads to apurinic sites in DNA and G→T transversions, contributing to genetic instability, mutations, and tumor progression [49,64,65].

Nitrosative DNA lesions and damage caused by NO can be repaired by cellular repair processes. However, global repair in CCA cells was reduced to 30% under NO production, contributing to DNA mutations [64].

It has been shown that CCA tissues were characterized by an upregulation of iNOS, which was inversely correlated with tumor differentiation and associated with poor prognosis, suggesting that iNOS may promote carcinogenesis [66]. Moreover, it has been reported that NO production, through iNOS, also induces the accumulation and activation of HIF-1α, which in turn upregulates iNOS, creating positive feedback that implements nitrative and oxidative DNA damage, resulting in tumor growth, invasion, and metastasis [67].

In this regard, higher iNOS expression and NO production seem to be related to CCA cells’ proliferation, vessel invasion, and lymph node metastasis. The process of metastasis requires the destruction of the extracellular matrix (ECM) as an essential initial step. Matrix metalloproteinases (MMPs) play a critical role in ECM degradation, and several reports have highlighted that iNOS positivity in iCCA tissues correlated with increased levels of MMP-2 or MMP-9. Thus, iNOS could promote iCCA invasiveness by increasing MMP expression. In this way, iNOS may be a useful biomarker to predict disease progression in iCCA [66].

**Figure 1:**
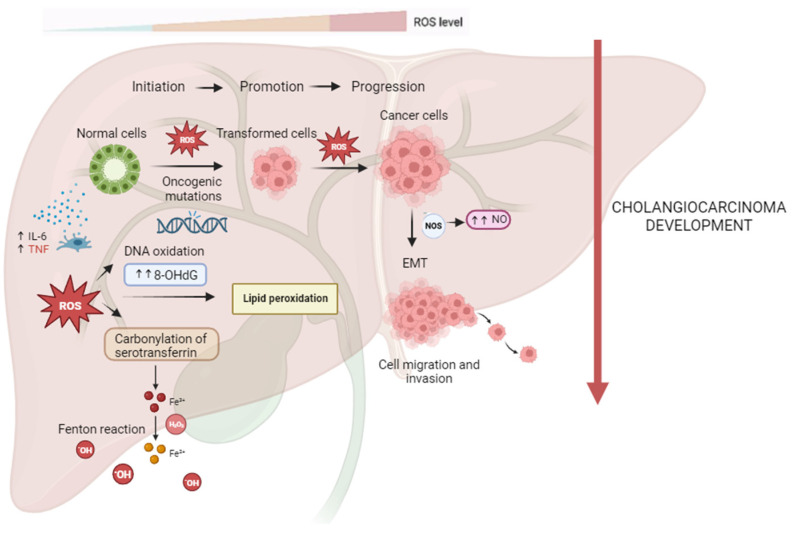
Reactive oxygen and nitrogen species (RONS) promote cholangiocarcinogenesis and progression. In the process of carcinogenesis, RONS can contribute to DNA damage with the formation of high levels of 8-ohdG, leading to oncogenic mutations and cell hyperproliferation. ROS-induced lipid peroxidation is responsible for DNA adducts and, thus, genomic instability. CCA tissues with ROS-mediated serotransferrin carbonylation show significantly higher Fe^3+^ concentrations, resulting in further generation of ROS through the Fenton reaction, which may contribute to all steps of CCA carcinogenesis. Higher iNOS expression and higher NO production may be related to CCA cells’ proliferation, vessel invasion, and lymph node metastasis of CCA via EMT.

CCA oxidative and nitrative damages are shown in Figure 1.

## 5. Redox Signaling in CCA

Increased ROS/RNS levels are involved in the regulation and disruption of redox signaling. Aberrant redox signaling may activate oncogenic pathways, promoting cancer cell survival, proliferation, invasion, angiogenesis, inhibition of apoptosis, and chemo- and radioresistance (Table 1).

The Keap1/Nrf2 pathway plays an important role in controlling antioxidant expression and protecting against drug toxicity and oxidative stress. The Keap1/Nrf2 pathway acts as a double-edged sword in cancer cells, and previous studies have demonstrated the involvement of this pathway in various cancers, including gallbladder and pancreatic cancer. While it plays a protective role in normal cells by eliciting anti-oxidative enzymes and preventing malignant transformation, cancer cells often take advantage of the Keap1/Nrf2 pathway to protect themselves against the oxidative stress induced by chemotherapy and support their growth. In CCA, the effect of this pathway on chemoresistance and prognosis is not fully clear. However, studies have indicated that unregulated Nrf2 provides resistance to anticancer drugs and reactive oxygen species, leading cancer cells to metabolic reprogramming.

Patients with dCCA exhibited a correlation between increased Nrf2 levels and poor prognosis, with Nrf2 overexpression emerging as a significant predictor of chemoresistance. Furthermore, Nrf2 has been identified as a potential factor influencing the efficacy of adjuvant chemotherapy following surgery [68].

Similar to Nrf2 signaling, the forkhead box O (FoxO) family has been proposed as a major mechanism in the cellular defense against oxidative stress. In particular, FoxO3, a transcriptional regulator of Keap1, participates in active crosstalk with Nrf2 signaling, regulating various cellular responses, including oxidative defense, proliferation, survival, tumorigenesis, and chemoresistance. In CCA cells, it has been shown that FoxO3 depletion correlates with enhanced tumorigenicity and drug resistance. FoxO3 depletion resulted in Keap1 downregulation, thereby activating Nrf2 and inducing cytoprotective and anabolic genes. FoxO3 silencing leads to a decrease in ROS production, protecting cells from oxidative stress-induced death in a Nrf2-dependent manner [69].

Recently, it has emerged that FoxO1, another member of the FoxO family, impairs autophagic flux in human CCA cells, leading to oxidative stress, mitochondrial dysfunction, and apoptosis, identifying this transcription factor as a potential therapeutic target for CCA treatment [70].

Recent studies have shown that persistent oxidative stress induces oncogenic transformation in cholangiocytes through downregulation of the tumor suppressor early B cell factor 1 (EBF1) and activation of ZNF423, resulting in CCA progression with poor prognosis.

EBF1 is involved in several developmental pathways, for example, in B cell differentiation, bone development, adipogenesis, retinal cell differentiation, and kidney development. In addition, EBF1 plays an important role in the differentiation of stem cells into mature cells [71]. EBF1 is believed to play suppressive roles in cancer promotion and progression, and recently, its downregulation has been found in many tumors. In CCA patients, the reduced expression of EBF1, accompanied by increased levels of 8-oxodG, is positively correlated with a poor prognosis [72].

ZNF423 is a transcription factor and an oxidative stress-responsive gene involved in the development and progression of CCA. ZNF423 was found to be overexpressed in CCA cells compared to normal bile duct cells adjacent to the tumor, and its expression positively correlated with 8-oxodG formation. Silencing of ZNF423 significantly inhibited cell proliferation and invasion of CCA cells, suggesting its oncogenic properties [73].

**Table 1:** Redox-altered expression profiles of oncogenes and tumor suppressor genes in CCA.

	Expression	Effects in CCA	Reference
HULC, H19	↑	Expression of inflammatory genes; enhanced tumorigenesis	[44]
Nrf2	↑	Malignant growth and chemoresistance	[68]
FoxO3	↓	Enhanced tumorigenicity and drug resistance	[69]
FoxO1	↑	Promotion of oxidative stress, mitochondrial dysfunction, and apoptosis	[70]
EBF1	↓	Cancer promotion and progression; enhanced oxidative stress damage	[72]
ZNF423	↑	Cell proliferation and invasion	[73]

## 6. *Clonorchis sinensis* and *Opistorchis viverrini* Mediated Redox Imbalance

The incidence of CCA is higher in Asiatic regions, where liver flukes (namely *Opisthorchis viverrini* and *Clonorchis sinensis*), diffused in these regions, represent well-established risk factors for this tumor [74,75,76].

With millions of infected persons, the liver fluke parasites *Clonorchis sinensis* and *Opistorchis viverrini* have a significant negative impact on public health; indeed, they cause chronic infection that induces hepatobiliary inflammation, leading eventually to CCA [77]. In a hamster model of *O. viverrini* associated CCA, it has been observed that after 12 weeks of infection with this parasite, the biliary epithelium is highly inflamed with increasing fibrosis that drives CCA onset [78].

The adult flukes and pathological alterations may work as a nidus for bacterial infection and the development of intrahepatic stones. Additionally, the liver flukes secrete metabolic products (referred to as ESPs), which are highly immunogenic and interact with the biliary epithelium or with it to promote proliferation, prevent apoptosis, and increase inflammation [79].

Therefore, the parasites induce tissue damage both by mechanical and chemical irritation and specific liver fluke immune responses.

During the *O. viverrini* infestation, NO is synthesized from L-arginine through iNOS by inflammatory cells in order to kill the parasite. N-nitrosoproline (NPRO) in urine, after proline loading, and plasma and urine nitrate levels have both been reported to significantly increase in *O. viverrini* infected subjects [80,81]. Additionally, N-nitrous dimethylamine (NDMA) was found in *O. viverrini* infected people’s urine and was linked to lymphoproliferative reactions to two parasites’ antigens [82]. CYP2A6, an enzyme that mediates NDMA cancerogenic activation, was induced in *O. viverrini* infected subjects [83,84]. These data suggest a key role of this parasite in promoting oxidative stress and cholangiocarcinogenesis.

Similarly, in an *O. viverrini* associated CCA hamster model, iNOS activity was detected during chronic inflammation, leading to DNA damage by 8-nitroguanine and 8-oxodG, the levels of which increased with the augment of infections [85,86]. Moreover, NO has also been reported to inhibit apoptosis downstream of cytochrome c [87]. All of these mechanisms facilitate carcinogenesis [88].

Infection by *O. viverrini* may even act through lipid peroxidation (LPO)-related oxidative DNA damage. It has been shown that etheno-DNA base adducts 1, N6-etheno-2′-deoxyadenosine (εdA) and 3, and N4-etheno-2′-deoxycytidine (εdC), markers for LPO-derived DNA damage, enhanced in an age-dependent manner from *O. viverrini* infection in a hamster model, likely as a result of DNA repair. Additionally, increased adduct production was associated with higher expression of alkyladenine DNA glycosylase (AAG), which excises εdA. The development of LPO-derived etheno-DNA adducts, if not fully repaired, may result in DNA lesion accumulation favoring carcinogenesis [47]. Along this line, the same authors demonstrated that εdA and εdC were present in white blood cells (WBC) and urine in *O. viverrini* infected subjects, and their levels decreased after antiparasitic therapy [48]. Therefore, following infection with this parasite, oxidative/nitrosative-related DNA damage can be decreased by antiparasitic treatment.

Moreover, in a hamster model infected with *O. viverrini*, iNOS expression was followed by HO-1 detection, the molecule implicated in protection against oxidative stress. HO-1, localized in Kupffer cells and bile duct epithelium, generated iron deposition, which can induce carcinogenesis by creating reactive oxygen molecules [89].

In a hamster model of CCA induced by *O. viverrini*, MMP-9 expression was linked to the development of peribiliary fibrosis together with the activation of Rac1 and iNOS, which may enhance DNA damage and cholangiocarcinogenesis [90].

In the RAW macrophage cell line, it was shown that treatment with an *O. viverrini* antigen induced the expression of TLR2, leading to an increase in NF-κB, iNOS, and COX-2 expression, suggesting a role of this parasite in promoting inflammation, nitrosative stress, and cholangiocarcinogenesis [91].

It has been reported that melatonin has a protective effect against *O. viverrini* induced oxidative and nitrosative DNA damage. Indeed, in a hamster model infected with *O. viverrini*, melatonin inhibited the expression of oxidant-generating genes such as iNOS and NF-kB and inflammatory factors (TNFα and IL1β) associated with an augment of antioxidant gene levels (such as Nrf2) [92].

The isoform CD44v of the cancer stem cell marker CD44 mediates the reactive oxygen species (ROS) defense through stabilizing xCT (a cystine–glutamate transporter) and inducing glutathione synthesis [93,94].

It has been observed in CCA tissues of a hamster model infected with *O. viverrini* a decrease in phospho-p38MAPK (a major ROS target) expression and instead an increase in CD44v levels during bile duct transformation. CCA patients (CCA dependent or not on O-viverrini infection) showing CD44v overexpression and negative p38MAPK phosphorylation had a significantly shorter survival rate than the ones showing low CD44v levels and positive p38MAPK phosphorylation.

Silenced CCA cells for CD44 displayed lower levels of xCT and glutathione, which caused high production of ROS. This, in turn, led to an enhanced p38MAPK activation. Furthermore, the cells treated with sulfasalazine, an xCT inhibitor, suppressed the ROS defense system by inhibiting GSH synthesis, driving a decrease in cell proliferation and stimulation of cell death.

The authors suggest that CD44-positive cells could represent the cell origin of CCA cancer stem cells. They can maintain redox homeostasis by increasing GSH production through the CD44v8–10–xCT system, resulting in evasion of cell death.

Thus, an xCT-targeting drug could improve CCA therapy by the sensitization of CD44-positive cells to the available drug by blocking the mechanism of the ROS defensive system [95].

Of interest, a Thioredoxin (Trx) has been detected in a cDNA library of *O. viverrini* [88]. It is released as an excretory–secretory product of the adult parasite picked up by the nearby epithelial cells [96]. OvTrx was able to inhibit apoptosis of bile duct epithelia induced with hydrogen peroxide by downregulating apoptotic genes and overexpressing antiapoptotic-related genes, suggesting that OvTrx blocked oxidative stress-induced apoptosis [97]. These last data indicate, therefore, a possible role of *O. viverrini* in even providing a defense against oxidative stress.

It is well known that also *C. sinensis* is closely associated with chronic inflammation and oxidative stress, which create a favorable microenvironment for the initiation and promotion of CCA [79].

Endogenous oxidative and nitrative DNA damage linked to *C. sinensis* infestation has been studied in both human and animal models [79]. Oxidative stress products such as 8-nitroguanine and 8-OxodG accumulate in chronic inflammatory areas around the bile duct epithelium via local NO generation triggered by iNOS. As a result, bile duct epithelial cells are continuously exposed to high amounts of oxidative damage, which promotes CCA initiation and development [85,98].

Human HuCCT1 CCA cells, exposed to *C. sinensis* ESPs, increased their free radical production via activation of NADPH oxidase (NOX), xanthine oxidase (XO), lipoxygenase (LO), COX, and iNOS, indicating an active role of *C. sinensis* excreted factors in modulating carcinogenesis [79]. Indeed, in a *C-sinensis* infected mouse model, COX-2, 5-LOX, and increased 8-OxodG were detected in the inflammatory areas [99]. In addition, NF-kB was overexpressed in ESP-exposed HuCCT1 cells, leading to the inhibition of peroxiredoxin 6 (Prdx6), an enzyme that reduces a wide spectrum of peroxides. This mechanism was, however, compensated by an increase in other ESP-induced redox-active transcription factors that bypassed NF-kB-mediated repression, driving ESP-induced Prdx6 expression. It was observed that the intensity and extension of immunoreactive NF-kB and Prdx6 staining in the *C. sinensis* infected mouse were associated with the severity of injury and infection duration, indicating that the increase in these factors may be linked to the persevering of hepatobiliary anomalies [100].

In a mouse infected with *C. sinensis*, liver fluke infestation enhanced the production of pro-inflammatory factors such as TNFα, IL1, and IL6, indicating that persistent upregulation of these cytokines is promutagenic for malignant cell transformation during chronic inflammation states [99]. Elevated levels of TNFα and IL6 caused activation of NF-kB, which, in turn, increased pro-inflammatory genes such as iNOS and IL6, resulting in inflammation boosting [101].

In the case of RNS, exposure of HCCA cells to ESPs of *C. sinensis* with NDMA induced proliferation in the G2/M phase and expression of cell-cycle-associated proteins, such as E2F1, cyclin B, and p-retinoblastoma (pRB) [102,103]. Mechanical and chemical irritation induced by *C. sinensis* and NDMA was believed to be the possible cause of genetic modifications driving neoplastic transformation by overexpression of the oncogene PSMD10 and cyclin-dependent kinase 4 gene CDK4 and downregulation of tumor suppressor gene p53 and protein retinoblastoma (RB) [104].

Interaction of *C. Sinensis* with other stromal cell redox balances has also been reported. Human hepatic stellate cell LX2 ferritin heavy chain, present in ESPs, induced the release of free radicals by the activation of NADPH oxidase, iNOS, and xanthine oxidase, leading to NF-kB activation [105].

*C. sinensis* ESPs contain myoglobin (Mb), a protein that has peroxidase activity and reduced H_2_O_2_ and NO levels in LPS-activated macrophages to escape from the host immune responses [106,107].

Ferroptosis is an iron-dependent, oxidative form of regulated cell death characterized by a deadly build-up of lipid peroxidation products and ROS. This condition is induced by several genes linked to iron metabolism and oxidative stress pathways and has been found to be involved in cancer development [53].

Studies on iron regulatory proteins and iron discrimination have been conducted in liver-fluke-associated CCAs. It has been found that iron is highly expressed in malignant tissues and that high iron build-up is linked to poor prognosis. In addition, transferrin receptor-1 expression augmented in cancer cells of cholangiocarcinoma tissues when compared to normal surrounding tissues and was significantly associated with cholangiocarcinoma metastasis. Indeed, the upregulation of transferrin receptor-1 expression was linked to an increase in the intracellular labile iron pool (LIP), leading to CCA progression with a critical outcome [108].

This evidence strongly suggests an active role of these liver flukes in promoting redox imbalance and carcinogenesis.

Importantly, patients with chronic PSC have elevated oxidative stress and dysregulated 9antioxidant responses [109], and murine models of cholestasis are characterized by oxidative stress markers, such as 4-HNE and MDA [110,111,112].

Recent data show elevated levels of Trx1 in PSC patients, which coincides with the inhibition of Thioredoxin-interacting protein (TxNIP), while the amount of mitochondrial Trx2, along with peroxiredoxins 3, 5, and 6, are significantly reduced. In addition, HO-1 and gamma-glutamylcysteine synthetase are suppressed, whereas the chaperone glucose-controlled protein 78 increases, indicating higher cellular stress.

These data indicate that, in addition to severe alterations in antioxidant responses, cholestasis influences both cytosolic/nuclear (Trx1) as well as mitochondrial (Trx2) redox pathways [113]. Therefore, for patients who develop cholangiocarcinoma from PSC, similar to what happens for worm-induced CCA, the altered redox system possibly represents a favorable scenario for the development of cancer.

## 7. Antioxidant Defenses in CCA

Cells have antioxidant defense systems to counterbalance the effects of oxidants. However, RONS accumulation may overload antioxidant systems, rendering them insufficient to protect cells from oxidative stress.

In CCA, a downregulation in several antioxidant genes has been described [114]. Total antioxidant status value was significantly lower in CCA patients’ serum samples compared to healthy controls (TAS), while total oxidant status (TOS) levels were significantly higher [115]. In particular, it has been shown a significant decrease in the expression of the SOD2 and CAT enzymes in human CCA tissues [116]. In line with these data, in CCA specimens, Mn-SOD expression was significantly lessened in cancer cells compared to the normal bile duct cells located at the tumor-adjacent areas [52]. Moreover, the activity of NAD(P)H:quinone oxidoreductase-1 (NQO1), a xenobiotic-metabolizing and antioxidant enzyme, is suppressed by inflammatory cytokines in cholangiocarcinoma cells, with the consequent increase in NO production and oxidative stress [117].

In contrast, an over-expression of some antioxidant enzymes such as Trx has been highlighted as a strategy to confer cytoprotection for cholangiocarcinoma cells against oxidative stress and induce cell resistance to anticancer agents [118,119].

Moreover, the levels of glutamate dehydrogenase (GDH), which produces a precursor to glutathione, were closely associated with CD34 positivity, cell differentiation, invasion, lymph node metastasis, and poor prognosis in eCCA patients. GDH could induce cell proliferation, migration, and invasion of eCCA cells through the TGF-β signaling pathway [120].

## 8. Possible CCA Treatments

As mentioned above, cholangiocarcinoma is a highly aggressive malignancy whose symptoms usually appear at a late stage when chemotherapy and radiotherapy are relatively ineffective. In addition, CCA is characterized by high multidrug resistance (MDR), which represents an obstacle to the improvement of therapeutic responses. Owing to this, CCA has a poor prognosis, and improved treatments for this tumor are, therefore, required. Changes in the redox signal are closely related to the growth and drug resistance of tumor cells, and thus, regulating the redox levels of tumor cells may be a strategy for the development of potential anticancer therapies [121].

Therefore, the evaluation of the possible benefits of a therapy based on antioxidants that aim to restore redox status could be an attractive strategy for the treatment of cancer. The literature data are controversial in this regard, and the benevolent and detrimental effects of antioxidants are much debated in clinical trials and cancer research [122].

The advantages and disadvantages of the use of antioxidants in cancer therapy are shown in Table 2.

A possible approach to overcome the MDR of CCA may be doxorubicin (DOX)-incorporated ChitoHISss nanoparticles, which have been constructed for pH- and redox-sensitive delivery in human cholangiocarcinoma cells. DOX-incorporated ChitoHISss nanoparticles were specifically delivered to tumor tissues, where they efficiently inhibited tumor growth, highlighting their promising role in CCA treatment [121].

Another study indicated the use of Niraparib, a poly(ADP-ribose) polymerase (PARP) inhibitor, as a potential therapy. It was shown that Niraparib induced a rapid increase in ROS levels accompanied by an augment in the phosphorylation of p38MAPK, rendering CCA cells more susceptible to drug treatment [126].

The screening of a redox library has identified Hinokitiol, a natural small molecule compound, as a candidate molecule capable of inhibiting the cancer stemness of iCCA cells by downregulating the expression of the ERK and P38 pathways. Moreover, its combination with the clinical drug Palbociclib showed significant antitumor effects [121].

Recent evidence focuses on the role of some food-derived polyphenols, which may exert antiproliferative effects on tumor growth. These compounds show their higher efficiency if used in addition to existing chemotherapeutic treatments, leading to cancer cell death by apoptosis or necrosis. For example, caffeic acid, a polyphenol extracted from the propolis of honeybee hives, exerts antiproliferative effects on cholangiocarcinoma and other tumor types in vitro and in vivo by inhibiting the nuclear factor-k-B pathway [127].

Moreover, xanthohumol (XN), a flavonoid of the hop plant, has been recently identified as an anti-inflammatory, antioxidant, and cancer chemopreventive molecule. It reduced ROS formation and iron accumulation, offering protection against DNA damage. XN effectively suppressed the growth of tumors and induced apoptosis in CCA cells and tumor-inoculated mice, inhibiting STAT3 activation and suppressing the Akt/NFκB signaling pathway [128].

Among the molecules with potential anticancer effects, the polyphenol resveratrol (3,4′-5-trihydroxy-trans-stilbene; RES) is one of the most promising. The literature data have indeed revealed that RES may inhibit the growth of leukemia, breast, and colorectal cancer cells, arresting the cell cycle in the S/G2 phase transition and altering the regulatory mechanisms of apoptosis and DNA synthesis [129]. Furthermore, it induces premature senescence via ROS-mediated DNA damage [130,131].

Concerning CCA, RES has been shown to have higher cytotoxic and pro-apoptotic effects on CCA cells [129]. In particular, it seems to decrease autophagic flux, leading to an increase in ROS levels and, thus, resulting in cell death [132]. In addition, the administration of low doses of RES increases cell sensitivity to chemotherapeutic agents both in vitro and in vivo, suggesting that RES treatment may be a potential therapy to improve CCA chemosensitivity [133].

However, other studies suggest that prophylactic properties of antioxidants have often revealed a failure and have even been found to increase cancer risk, thus highlighting more complex regulatory pathways modulating oxidative stress in normal as well as in neoplastic cells. Oncogenic signals acquired during malignant transformation might both induce ROS generation and hence stimulate cell proliferation through redox-sensitive transcription factors and, at the same time, promote adaptive antioxidant upregulation to minimize oxidative damage and counteract potential toxic effects.

It has been, therefore, proposed a combination regimen composed of a combination of cytostatic drugs, in addition to agents such as the curcumin analog EF24, that reduce the antioxidative response, overcoming resistance and enhancing therapeutic selectivity against cancer cells. In particular, EF24 inhibits proliferation, migration, and clonogenicity, leading to apoptosis by increasing oxidative stress in cholangiocarcinoma cells: it increases ROS levels and, at the same time, depletes cells of GSH [134,135].

**Figure 2:**
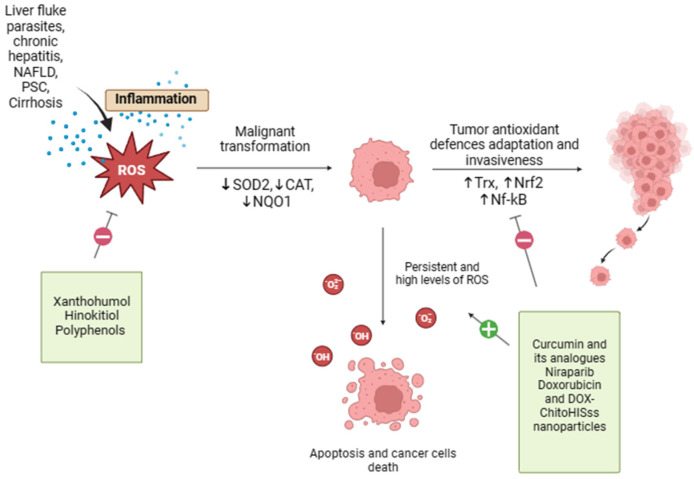
Oxidative stress and possible therapeutic approach in CCA. Oxidative stress and redox homeostasis are implicated in all phases of cholangiocarcinogenesis, and the regulation of tumor cell redox levels may be a potential strategy for the development of anticancer therapies. Xanthohumol, Hinokitiol, and some food-derived polyphenols exert antioxidant properties that may reduce ROS formation, offering protection against oxidative damage. On the other end, curcumin, resveratrol, doxorubicin, and DOX-ChitoHISss nanoparticles inhibit tumor antioxidant system adaptations and sensitize cholangiocarcinoma cells to chemotherapeutic treatment, leading to increased oxidative stress and cancer cell death.

Oxidative stress and possible therapeutic approach in CCA are shown in Figure 2.

## 9. Conclusions

In conclusion, CCA stands as a formidable adversary within the landscape of oncology. It is characterized by late-stage diagnoses, limited treatment options, and the vexing challenge of multidrug resistance. As our understanding of CCA deepens, oxidative stress and redox signaling emerge as pivotal players in its pathogenesis, shedding light on novel avenues for comprehending and addressing this complex malignancy. The intricate interplay between oxidative stress and antioxidant defense represents an intriguing aspect of CCA’s etiology. It influences the molecular landscape of CCA by fostering genetic instability, inflicting DNA and protein damage, and inducing lipid peroxidation. These processes collectively contribute to the initiation and progression of CCA.

The outlook for CCA treatment has shown promise through innovative therapeutic strategies. Drug-incorporated nanoparticles, PARP inhibitors, small molecule compounds such as Hinokitiol, and polyphenols derived from food sources hold the potential to augment treatment efficacy. They offer a potential strategy for improving the prognosis of CCA patients, providing new paths toward enhanced treatment outcomes. However, the role of antioxidants in the context of CCA therapy remains multifaceted. This complexity necessitates ongoing and meticulous research to untangle the intricate web of potential benefits and challenges that antioxidants present. As antioxidants navigate this intricate landscape, a balanced approach becomes paramount, considering the delicate equilibrium between oxidative stress and antioxidant defense. In this equilibrium lies the key to unlocking the full potential of antioxidants in CCA treatment.

Looking forward, a deeper understanding of these mechanisms kindles hope for more effective therapeutic approaches for CCA treatment.

## Figures and Tables

**Table 2:** Advantages and disadvantages of the use of antioxidants in cancer therapy.

	Advantages	Disadvantages	Reference
Antioxidants	Widely available	Difficulty in achieving the effective in vivo concentration	[123,124]
	Short-lived and fast-acting oxidizing biomolecules	Antioxidants may promote cancers rather than cure them	[125]
	Neutralization of free radicals’ excess	Cancer cells may benefit from the use of antioxidants	[125]
	Enhancement of host antitumor immunity	Difficult to deliver to tumor mass	[124]

## Data Availability

No new data were created or analyzed in this study. Data sharing is not applicable to this article.
